# Automated information sharing in foster care: perspectives on impact and expansion

**DOI:** 10.3389/fped.2025.1543076

**Published:** 2025-05-26

**Authors:** Nathan M. Lutz, Mary V. Greiner, Lisa Vaughn, Amanda Schondelmeyer, Elizabeth Freehling, Katie Fox, Sarah J. Beal

**Affiliations:** ^1^ Division of General and Community Pediatrics, Cincinnati Children’s Hospital Medical Center, Cincinnati, OH, United States; ^2^ Division of Behavioral Medicine and Clinical Psychology, Cincinnati Children’s Hospital Medical Center, Cincinnati, OH, United States; ^3^ Department of Pediatrics, University of Cincinnati College of Medicine, Cincinnati, OH, United States; ^4^ Psychology Department, Eastern Michigan University, Ypsilanti, MI, United States

**Keywords:** foster care, information-exchange platforms, caregiver perspectives, healthcare staff perspectives, child welfare

## Abstract

**Introduction:**

For youth in foster care, healthcare information is often not communicated to relevant individuals, including foster caregivers and healthcare staff. Technology solutions designed to bridge that gap have been shown to increase information available, decrease time spent searching for information, and improve access to services. The feasibility of technology-based information sharing has been demonstrated with caseworkers.

**Methods:**

This qualitative study builds upon existing knowledge about the benefits of technology-based information exchange systems, utilizing interviews with healthcare staff (*n* = 41) and foster caregivers (*n* = 7). The purpose of this work is to understand their perspectives related to the utility of information-exchange technology, barriers faced, and potential improvements.

**Results:**

Three themes were identified: (1) Impact and Benefits: The high-yield benefits of automated, on-demand information-sharing platforms for children in foster care; (2) User Experience and Efficiency: Streamlined workflows and improved outcomes; and (3) Opportunities for Platform Expansion: How information sharing can be further improved. Healthcare staff and caregivers alike said using technology to share health and child welfare information was efficient and easy in a system that is fragmented. They also identified opportunities for expansion, such as including more sources of information (e.g., information from caregivers themselves).

**Discussion:**

These findings add to the evidence that information exchange platforms in child welfare increase the capacity of healthcare staff and caregivers while decreasing workload in an otherwise overburdened system. Expanding access to and use of information-exchange platforms in healthcare settings that serve youth in foster care may support the workforce who serve these children.

## Introduction

An estimated 200,000 children enter foster care (i.e., in child welfare custody and placed in out-of-home care) in the United States every year, with approximately 400,000 children in foster care at any given time (1). Foster care is disruptive to a child's health in multiple ways, including altering where and from whom children receive healthcare (2, 3). To effectively deliver healthcare services to children, it is important for healthcare staff to understand children's contextual factors [e.g., living situations, health histories, etc.; (4)] and for caregivers (e.g., foster parents, kinship care providers, group home staff) and child welfare professionals (e.g., caseworkers, guardians ad litem, court appointed special advocates) to understand children's health needs and recommendations from healthcare providers (4). For children entering foster care, this information may be initially communicated to child welfare professionals, who are responsible for relaying relevant information to healthcare staff and caregivers. Once children are in foster care, however, caregivers receive information from healthcare staff and relay that information to child welfare professionals. These variable processes, combined with caseworker turnover (5), uncertainty around information-sharing policies, and missing information (4), contribute to health information not being communicated (6, 7). Caregivers frequently report limited knowledge about the health histories of children placed in their care (6), while healthcare staff identify a lack of information about children's social history and failure to follow through with recommended care as challenges to working with the child welfare system (4).

Previous work demonstrates that technology solutions can address challenges in access to information about children's health when they are in foster care (8). The Integrated Data Environment to eNhance ouTcomes In custody Youth [IDENTITY] platform (9) is one such solution. This platform integrates administrative child welfare information with the electronic health record in real time to facilitate information sharing across systems. The benefits of IDENTITY to young people in foster care have been outlined in previous work (9), and IDENTITY was demonstrated to decrease time required to gather information, improve access to services, and ensure recovery of costs for services (10). Additionally, IDENTITY use was shown to be feasible for caseworkers (7). However, the perspectives of healthcare staff and caregivers who may also benefit from health information sharing when children are in foster care have not been described.

This qualitative interview study explores perspectives of healthcare staff and foster caregivers regarding accessing IDENTITY, the utility of information sharing, barriers to use, and how data sharing can be improved.

## Methods

### Setting

This study occurred at a freestanding pediatric medical center in the Midwest United States in its associated outpatient specialty clinic for young people in foster care. The medical center's Institutional Review Board and the children's services office approved this study (IRB #2022-0339 and #2022-0536). The study team was comprised of individuals with clinical (NL, MG) and research (NL, SB, MG, EF, KF) experience working with foster youth and caregivers as well as individuals with qualitative methodologic experience (LV, AS). Research team members (SB, MG) were involved in the creation of IDENTITY, the software that served as the focus of this qualitative study. Additionally, these team members are the medical (MG) and scientific (SB) directors of a center that serves youth in foster care within the medical center. The research team has conducted several studies that involve populations associated with the child welfare system (e.g., youth in foster care, foster caregivers, kinship care providers, caseworkers, state child welfare agencies), many of which have included qualitative methods or strategies like using advisory boards to inform study design. To enhance reflexivity, the team met weekly to discuss this project and related studies, reflecting on how findings from previous work, clinical experience, and input given from community partners shaped our interpretation of data for this study.

The medical center links child welfare and electronic health records (EHR) data in near real-time for caseworkers and healthcare staff to access, updated daily via IDENTITY. For details about the IDENTITY application, such as information sources, access, information updates, privacy-related considerations, and a screenshot of the user interface, see Greiner et al. (9). IDENTITY is used to create a flag in the EHR to indicate foster care status, viewable by healthcare staff. The flag is removed within 24 h of a child's exit from foster care.

### Study design

This descriptive qualitative study used interviews to assess the perspectives of healthcare staff and licensed foster caregivers and to provide a more comprehensive and unifying examination of information sharing when children are in foster care. Information on the participants and corresponding procedures is summarized below. For full details, see Supplementary Materials.

#### Healthcare staff participants

Healthcare staff (e.g., social workers, financial advocates, physicians) who had contact with a patient with an active EHR flag indicating the patient was in foster care were eligible for this study. A purposive, stratified sampling approach (11) was used to identify potential participants with a range of experience using IDENTITY. The study team generated a report of individuals outside of the foster care clinic who utilize IDENTITY frequently (i.e., >50% of eligible encounters) and a report of non-users who had contact with foster youth (>6 encounters in 6 months) but did not utilize IDENTITY (<10% of eligible encounters). The study team identified and contacted 217 eligible individuals via email. Forty-one professionals (22 users and 19 non-users of IDENTITY; 19% of those invited) agreed to participate in an interview. Enrolled participants (*N* = 41; Table 1) were between 25 and 67 years of age (M = 41.28, SD = 10.39), primarily White, non-Hispanic (*n* = 31, 76%), and predominantly female (*n* = 36, 88%) and had been employed at the institution where the study took place for an average of 9 years across various healthcare roles (e.g., social worker, clinical provider, administrative support, patient services).

**Table 1:** Descriptive characteristics of study participants.

Healthcare staff perspectives		
Variable	Mean (SD)	*N* (%)
IDENTITY non-users	–	22 (54%)
IDENTITY users	–	19 (46%)
Role, administrative	–	3 (7%)
Role, patient services	–	7 (17%)
Role, provider	–	14 (34%)
Role, social work	–	17 (41%)
Number of years at institution	9.1 (6.0)	–
Age, Years	41.3 (10.4)	–
Gender, Female	–	36 (88%)
Gender, Male	–	5 (12%)
Race, Black/African American	–	7 (17%)
Race, White or Caucasian	–	31 (76%)
Race, More than one race	–	2 (5%)
Race, Did not disclose	–	1 (2%)
Ethnicity, Non-Hispanic	–	39 (95%)
Ethnicity, Hispanic	–	2 (5%)
Foster caregiver perspectives		
Variable	Mean (SD)	*N* (%)
Index child age (years)	2.7 (1.7)	–
Index child time in placement (days)	249.1 (171.9)	–
Caregiver age (years)	41.4 (9.8)	–
Caregiver gender (female)	–	7 (100%)
Caregiver race and ethnicity (White, Hispanic)	–	1 (14%)
Caregiver race and ethnicity (White, Non-Hispanic)	–	6 (86%)
Years as a foster parent	10.3 (11.6)	–
Total foster youth cared for	26.6 (34.9)	–
Number of current foster children	1.9 (1.2)	–
Number of own children	3.4 (2.6)	–
Number of children under 18	2.3 (1.4)	–
Number of biological, adopted, or stepchildren under the age of 18 living in the home	2.7 (1.9)	–
Number of other adults in the home	1.1 (0.7)	–
Caregiver had some health information before IDENTITY, but could use more	–	5 (71%)
Caregiver had all the health information they needed before IDENTITY	–	2 (29%)

#### Healthcare staff procedures and materials

A form was sent via email to eligible participants using Research Electronic Data Capture [REDCap; (12)] survey software where they could indicate interest in participating. A research coordinator contacted interested participants to schedule interviews using a video conference platform (Microsoft Teams). Participants gave verbal consent and completed a brief demographics survey followed by a recorded interview. The interviewer used one of two semi-structured guides: one for IDENTITY users and another for non-users. Users were asked how they learned about IDENTITY, when and how they use it, its impact on their work, and what challenges or barriers they face. While exact numbers related to frequency of use were not provided, users estimated they used IDENTITY anywhere from every few months to every time they get a referral. Non-users viewed a demonstration version of IDENTITY and were asked questions about the potential impact of the platform as well as how they obtain child welfare information without IDENTITY.

Interviews were conducted between October 2022 and April 2023. We continued conducting interviews until we identified informational redundancy, commonly referred to as saturation [i.e., the team continued to review transcripts until no additional codes arose, both within subject types (healthcare staff, caregivers) and across the group], guided by Saunders et al. (13), Mason (14), and Malterud et al. (15). All interviews were conducted by the same trained interviewers (EF, MG). Interviews lasted approximately 30 min. Verbatim transcripts of the interviews were used for analysis. Participants were compensated with a $50 gift card.

#### Foster caregivers participants

Adult caregivers (e.g., licensed foster parents, approved relative or non-relative kinship providers, group home staff, and independent living workers) of children in foster care received invitations to participate via caseworkers. Eight participants expressed interest, all of whom were licensed foster caregivers (i.e., none of the kinship providers, group home staff, or independent living workers expressed interest). One could not schedule an interview and was not enrolled in the study.

Enrolled participants (*N* = 7; Table 1) were between 32 and 59 years of age (M = 41.4, SD = 9.8), primarily White and non-Hispanic (*n* = 6, 86%) and female. Experience among foster caregivers varied [licensed between <1 and 30 years [M = 10.3, SD = 11.6] and having cared for between 1 and 89 children [M = 26.6, SD = 34.9]]. Participants answered interview questions with respect to an index child in the custody of the county that approved the study. If the participant had more than one foster child at the time of the baseline interview, the participant could choose the index child. Index children were <1–5 years of age (M = 2.7, SD = 1.7) and had been placed in the home between 84 and 607 days (M = 249.1, SD = 171.9). To our knowledge, all index children remained in their placement throughout the duration of the study.

#### Foster caregivers procedures and materials

Eligible participants were given the opportunity to indicate interest via a REDCap survey distributed by the county child welfare agency. Interested participants were contacted by research staff to schedule a recorded interview on Microsoft Teams. Characteristics about the children in the participant's home were extracted from IDENTITY and included age and length of stay in placement. Participants provided electronic informed consent and completed a brief demographics survey at the start of the recorded interview. Participants were asked how much information they had about the foster child in their care, including what health information they were given at the time of placement, what they learned since placement and how they learned it, whether the information was accurate and complete, and how missing or inaccurate information impacted the care they sought for the foster child.

Participants were then shown a live demonstration of IDENTITY and were allowed to review their foster child's record. After reviewing the information, participants were asked if it was accurate and complete, how easy or hard it was to find information, whether any information was new to them, and how having access to information earlier in the child's placement would have been useful. Participants were also asked if and how they would use IDENTITY in the future and what other features they would like. After the baseline interview, participants were given access to the IDENTITY records for their foster children, which they could view at any time until the child left the placement, at which time their access was terminated.

One month later, participants completed a follow-up interview. During this interview, participants were asked how and when they used IDENTITY, what information was useful, what information was missing, and what information was not available in IDENTITY but could be found in other sources. Participants were also asked what additional information about the foster child (e.g., child's preferences or other information) would have been useful early during placement and what information they would be willing to provide through IDENTITY if they could do so.

Data collection began in June 2022, when the first participant completed the study interest survey, and ended in November 2022 when the last participant completed the follow-up interview. All interviews were conducted by the same trained interviewers (EF, SB). Baseline interviews lasted approximately 60 min, and follow-up interviews last approximately 45 min. Participants were compensated $40 for the baseline interview and $20 for the follow-up interview using a reloadable debit card. Verbatim transcripts of the interviews were used for analysis, and data was stored in REDCap.

### Data analysis

All data were de-identified prior to analysis. The coding team (EF, MP, HS, KR, HD, HG, JP) used an inductive thematic analysis approach (16–19) to analyze the interview transcripts. Codes were generated based on recurring patterns and significant statements that arose during interviews. The team identified these concepts as interviews were being conducted and added to the codebook as the study progressed. The study team met to discuss and refine codes to maximize consistency and coherence. Themes were finalized through iterative discussions. Transcripts from healthcare staff (*n* = 41 from 22 IDENTITY users and 19 non-users) and foster caregivers (*n* = 13 from 7 initial interviews, 6 follow-up interviews) were included and coded separately, applying the process described below. The coding team was composed of staff members with qualitative coding expertise and members of the study team with expertise in the care of foster youth. Each member read several transcripts to become familiar with the data. Team members worked together to generate initial codes for a preliminary codebook derived from 9 transcripts. The remaining transcripts were each coded by two members. All coders met together regularly to discuss the codes, resolve disagreements, and revise the codebook until saturation was reached (14, 15). After coding all transcripts, the coding team sorted the codes into categories that captured patterns in the data. The categories were then distilled into overarching themes addressing the research question. Interview transcript data management and analysis was supported by Dedoose (20), a qualitative data analysis software. Participant characteristics data management and analysis used SAS 9.4.

## Results

We identified the same three themes from healthcare staff and foster caregivers: (1) Impact and Benefits, (2) User Experience and Efficiency, and (3) Opportunities for Platform Expansion.

### Theme 1. Impact and benefits: the high-yield benefits of automated and on-demand information-sharing platforms for children in foster care

Foster caregivers described challenges accessing healthcare information for children placed with them and a lack of awareness of how to obtain information. Some heard about historical health information after the child had been placed with them. Caregivers frequently reported feeling like detectives or as if they were piecing together a puzzle about the child's health history.

For a period of time, I was actually given access to.. some of her MyChart information.. I remember she had gone to the ER like a handful of time, more than I would have anticipated, you know, more than any of my children had ever been in such a short period of time. So that was kind of a red flag to me that she had been in the ER quite a few times. Umm, so that was the initial health information that I learned. (104—Foster Caregiver)

Similarly, healthcare staff who were non-users of IDENTITY described having to rely on multiple information sources (e.g., reading EHR chart notes, calling child protection hotlines) with inconsistent success resulting from those efforts. Healthcare staff who were IDENTITY users reported that many of those challenges were resolved because important information was available to them.

[IDENTITY] explained about why they are in the middle of a court case, who their foster parents are, how many placements there’s been. There’s even follow-up with medical appointments, too. So, everything was gonna be in that one location, which I thought was great. (115—Healthcare staff user)

IDENTITY also provided foster caregivers with children’s services and Medicaid information that had previously been difficult to access.

Yeah, I can just say that would be so helpful. Oh my God, it’s been a year and a half, and I have yet to receive an insurance card, so there’s that. […] I loved the caseworkers. I loved the phone numbers. I, I mean, I don’t even know who my kids’ supervisors are. So, I mean, that’s great to be able to access that. (108—Foster Caregiver)

Healthcare staff reported that IDENTITY provided information about patients that they previously didn't have access to.

I didn't know that you could see their siblings through [IDENTITY]. So that was helpful to see that or to realize that you could search by foster home to see the other kids in the home. (209—Healthcare staff non-user)

When asked to provide examples of times they used IDENTITY during the follow-up interviews, caregivers reported varied use, from looking at information in the program once (*n* = 1) to using it several times a week over the month between interviews (*n* = 3). Healthcare staff use also varied, from two or three times total (*n* = 2) to every patient in foster care (*n* = 8) or somewhere in between (*n* = 6). With this variety of uses also came varying degrees of awareness about IDENTITY functions to support caregivers and healthcare staff. As both groups became more familiar with the platform, they were eager to share it with others.

I think IDENTITY operates within its space and does great work. […] I think a brand ambassador that can come in the clinics every once in a while, like ‘hey, I'm here, you have any questions? Let me show you how.’ (105—Healthcare staff user)

We've certainly shared it as a resource with residents as well. Through my work with the [Medication Reconciliation] folks, I also shared it with some pharmacists who have no idea it exists. Then we're really excited to hear about it. So, I think just telling as many people as you can about it because I think it’s a great tool. (106—Healthcare staff user)

That’s awesome.. everybody’s really excited. I mean, when we talked about [IDENTITY] just to our foster parent group, everybody’s like, thank goodness, we just need this. (103—Foster Caregiver)

While the two groups were aligned in the view that IDENTITY was impactful, the type of information they referenced in the interviews differed. Healthcare staff typically used IDENTITY to learn more about the home environment and the child's placement, while foster caregivers used IDENTITY to learn more about health histories and contact information for both medical and child welfare staff.

### Theme 2: user experience and efficiency: streamlined workflows and improved outcomes

Participants found IDENTITY's user-friendliness and ease of access allows providers to spend more time focusing on the quality of care and follow-through for children.

I couldn’t imagine having to just, like, get all of the information from a foster parent who also isn't always privy to everything. And then kids can over exaggerate things—kids more often under exaggerate things, and will often like protect their family members, and so not knowing exactly what was going on, [IDENTITY is] very helpful, and I think it’s presented in a very non-biased way which is good. (107- Healthcare staff user)

I thought it was very simple to use. It’s not terribly complicated that there’s so much information. (103—Foster Caregiver)

Overall, IDENTITY users expressed satisfaction with the experience. For instance, an IDENTITY healthcare staff user (191) describes the efficiency of using IDENTITY.

[IDENTITY] just makes it faster. If I’m trying to figure out who the caseworker is or the correct caseworker is, I know I can sign on there as long as they’re in the appropriate county. I know I have access to that instead of calling children’s services, being on hold for a long period of time, or contacting a caseworker that had the case, you know, six weeks ago. And now we've moved on and they're not going to respond to me. So, I’ve wasted time. I feel like it just helps me save time by being able to access, even just the very simplest of information quickly. (191—Healthcare staff user)

Similarly, a caregiver (102) described getting updated information more quickly with IDENTITY.

I like the fact that you can look up and you can see, like, what their doctor’s office is and the phone number just in case you don't have that, the caseworker, because these caseworkers are ever-changing. Umm, so we don't always get updated, but it is updated there. Like I found out their new caseworker before they actually told me from IDENTITY because they put it in that way vs. I didn't even know what her name was. Like, so having that information, I think, is really useful. (102—Foster Caregiver)

While most interviewees found IDENTITY easy to use and efficient, some interviewees shared that their knowledge of the child's health history and IDENTITY were not always consistent. Sometimes people felt frustrated when they found out-of-date information.

Some of this stuff in Epic in the patient charts is very outdated, it’s not accurate. And I don’t know who determines when a note goes out. Like you’ll see a note that says, ‘per so and so this is this 1.18.2020’. Is it still valid? (199—Healthcare staff non-user)

Those shots are still not in there, and those were done in September. So those should have been added by now and they're still not in there. (103—Foster Caregiver)

### Theme 3: opportunities for platform expansion: improved program utility

Users employed IDENTITY as a resource for finding contact information and patient history information. Participants expressed a desire to expand the scope of IDENTITY, incorporating more healthcare systems, counties, and states; more sectors of information, such as education; and more comprehensive and up-to-date contact information.

You know what would be helpful is a copy of their medical [insurance] card. Because so many times the youth don't have it. I know there’s some super convoluted way that we can print it out from Epic if it’s been scanned, but if that could be scanned in [to IDENTITY], then we can be an access point. (140—Healthcare staff user)

The thing that it lacks is obviously connection with [non-medical center affiliated] pediatrician offices because I take him to the pediatrician pretty regularly and so all of that information is not in there. Only what essentially the foster care clinic reports he went there once. (107—Foster Caregiver)

Caregivers and healthcare staff requested more expansive information pertaining to child, family of origin, provider, and foster caregiver goals, medical history, safety plans, court dates, and child behavioral issues to improve the child's care as well as printable documents related to the child.

I really think it would be helpful information that rarely gets shared with us is—what the overall goal of a placement is in there, like the goal is reunification—but if I actually saw the goals that they had set for families to work on towards reunification, I think that would be helpful because sometimes they really need to be specific to that medical condition, to diabetes. Just kind of knowing what progress they're making or whether or not that’s even a consideration as part of reunification case plan goals. These are the things that we're asking the families to do. That would be helpful. (164—Healthcare staff user)

So that would be helpful because, like, kids that I've taken for evaluations at the preschool, they need documentation and they actually need the court paper. But I don't know how, would they take this as documentation as to what school district is responsible? Because by the time you track it down, it just takes a long time. And if you could print that and take it there, it just would cut out a lot of time. (103—Foster Caregiver)

Participants suggested additional design features including a message box or communication board to facilitate communication. Current users emphasized patient and case information details, while non-users hoped to streamline the use of IDENTITY through features such as a dashboard or patient snapshot to summarize important information. One healthcare staff user (140) captured the improved outcomes that would come from an expansion of IDENTITY to include contact information for all healthcare team members.

I think it would improve communication more.. if I, as a care manager, assigned myself to the care team. So, if a caseworker could get in and see my name and contact to know to contact me with questions, I think that would probably be helpful. Putting the care team into IDENTITY. (140—Healthcare staff user)

Consistent with this idea, caregivers also identified benefits that could be achieved if IDENTITY had additional sources of data.

Like, if there was a place that I could add info in about this child’s schedule preferences, fears, triggers, all of those things would be super helpful. (105—Foster Caregiver)

Both healthcare staff and foster caregivers were interested in building additional features into the IDENTITY platform, but they had slightly different priorities for expansion. Healthcare staff prioritized ways to increase their involvement in a child's care, while foster caregivers wanted to be able to provide information that would not be captured by the EHR or in child welfare agency data, bringing their own unique knowledge of the child's day-to-day experience as an additional source of data.

## Discussion

Poor information sharing contributes to poor healthcare outcomes for children in foster care. Information-sharing platforms are feasible solutions that contribute to meaningful improvements in experience and workload of child welfare professionals (7, 9, 10). This study adds to prior literature by exploring the user experience for both healthcare staff and foster caregivers, who described similar experiences with information sharing in this study. Both groups shared excitement, stating that, without IDENTITY, health information about children in foster care was often fragmented, had to be collected from many different sources, and was typically incomplete. IDENTITY was seen as more efficient and provided a history that they otherwise would not have been able to obtain.

Healthcare staff and foster caregivers alike noted the simplicity and ease of use. Both groups also identified the opportunity for improved communication within IDENTITY, but their goals varied. Healthcare staff users saw an opportunity to communicate between team members, such as care managers. Foster caregivers hoped to utilize IDENTITY to communicate about a child's non-medical needs, such as trauma triggers, food preferences, and comfort items. Both ideas would increase the complexity of IDENTITY but would provide for a more comprehensive picture of the child's needs. Different user experiences within a data-sharing system like IDENTITY may be necessary for different types of users. A foster caregiver portal, for example, might share non-medical information with other caregivers, like respite providers.

Along with their enthusiasm, users identified opportunities to improve the data-sharing system, particularly recommending the inclusion of data from additional healthcare organizations as well as other sectors, such as education. Children in foster care often have fragmented care, and it is quite common to have healthcare encounters at multiple institutions. To maximize its utility and the benefits provided, IDENTITY and other information sharing systems must be able to incorporate additional data sources.

### Implications of findings in the context of prior work

There are several important implications of these findings, which expand our current knowledge and practice about healthcare and child welfare information integration and sharing and provide insights regarding the future expansion of this work. First, findings indicated that professionals [healthcare providers in this study, child welfare professionals in other studies; (7)] and caregivers benefitted similarly from the same information-sharing platform. While technology is often either designed to focus on the healthcare provider [e.g., Epic; (22)], or the patient [e.g., MyChart; (23)], IDENTITY was able to deliver benefits to caregivers without intentional customization for that user base. While additional modifications (e.g., the opportunity for direct data input and expanded security logic to mask sensitive data) are needed prior to widespread distribution to caregivers, these findings indicate minimal work is likely required for caregivers to benefit from information exchange platforms like the one used in this study.

Second, the benefits to efficiency were achieved when data sharing was timely (i.e., daily data updates) and did not require dual data entry. This is a departure from other solutions on the market in child welfare and healthcare; future research may need to explore the trade-offs to efficiency if full and regularly updated automation cannot be achieved.

Finally, this study's focus on caregivers and healthcare providers is somewhat unique in a child welfare topic area, where the child welfare workforce and the youth/family of origin are most often the topic of focus for technology enhancement (8, 23). Notably, the work of evaluating IDENTITY is more developed with child welfare professionals, followed by healthcare staff—this likely reflects the power and resource distribution of child welfare funding. Leveraging benefits to other groups (e.g., healthcare providers, caregivers) may provide a more diverse funding stream to support technology advancement and sustainability.

There are important limitations to this study. Healthcare staff were selected based on high or low utilization of IDENTITY and selected to represent a broad variety of roles, but they may not represent all current and potential users. Foster caregivers were also selected from one county and may not represent other common caregivers for children in foster care, including kinship caregivers and group home staff. These types of users may have different needs for information sharing. Finally, the perspectives of youth in foster care themselves were not reflected in this study and are a necessary voice in determining all impacts of automated data sharing on outcomes.

This study identified important themes in the role of information sharing for healthcare staff and foster caregivers. Foster caregivers have historically been excluded from most patient-facing electronic health portals because it has been technically difficult to figure out how to regulate their access (e.g., when a placement change occurs). Data sharing systems like IDENTITY that use child welfare data to automatically regulate access for foster caregivers when they have a child in their home, highlight how these systems may address child welfare workforce shortages and ensure caregivers and healthcare providers have the information they need to effectively care for children in foster care. Additional research is needed to understand the full impact of data sharing on foster care outcomes, such as healthcare utilization and health status, and this work should include the voices of youth in foster care themselves. Broadly, this study suggests that automated data sharing is important for healthcare staff and foster caregivers as key stakeholders who seek to improve outcomes for children in foster care.

## Data Availability

The datasets presented in this article are not readily available because qualitative interviews were used for this study, and themes were generated from de-identified transcripts. Requests to access the datasets should be directed to Sarah Beal; sarah.beal@cchmc.org.
