# Supplementary material for: Automated information sharing in foster care: perspectives on impact and expansion
Source: Front Pediatr. 2025 May 26;13:1543076. doi: 10.3389/fped.2025.1543076 (PMC12146271; doi:10.3389/fped.2025.1543076)
Supplement: Supplementary file 1 [file Supplementaryfile1.docx]

# Supplemental Procedures

For both healthcare staff and foster caregivers, the interviews began with either verbal (healthcare staff) or electronic informed (foster caregivers) consent, followed by a brief demographic survey via REDCap. For both groups, interview questions were developed by expert review and informed by Kruchten’s (12) conceptual model of software development (see Supplemental Table 1 for the interview guide).

## Healthcare Staff Perspectives

### Recruitment and Consent Procedures

The Research Informatics department at the medical center, which manages IDENTITY, provided the study team with a list of IDENTITY users and non-users based on historical clinical data where care was delivered to a patient with an active flag in Epic that would alert healthcare staff that the child had an associated IDENTITY record. The investigators created a link to a study interest survey and sent it to eligible participants using REDCap. A research coordinator contacted participants who completed the REDCap interest form via phone or email to provide further information about the study.

### Qualitative Interview Procedures

Demographic survey items completed at the beginning of the interview included age in years, gender, race, ethnicity, current professional role, and number of years employed at the medical center. The study team utilized a semi-structured guide to allow for flexibility in the phrasing and delivery of questions to participants.

## Foster Caregiver Perspectives

***Recruitment and Consent Procedures***

The research team created a link to a study interest survey using REDCap, and the survey was distributed to eligible participants by the county child welfare agency. Any type of caregiver was eligible; only foster caregivers responded to the survey link indicating interest. Research staff contacted interested foster caregivers via phone or email to provide more information about the study and to schedule the virtual baseline interview.

### Qualitative Interview Procedures

Survey items completed at the beginning of the interview included demographic information (i.e., age, gender, race, and ethnicity), questions about their history as a foster caregiver (i.e., total number of years as a foster caregiver, total number of foster youth cared for in that time, and number of foster children currently in the home), and details about other family or household members (i.e., number of participants’ own children, number of participants’ own children under the age of 18 living in the home, and number of other adults living in the home). A semi-structured interview guide was developed and used by the study team.

Supplemental Table 1

Qualitative Interview Guides

| **Healthcare Staff Interview – IDENTITY User** |
| --- |
| 1. Can you tell me a little about your current position/role at [Institution name]? What does a typical day look like for you? |
| 2. Can you tell me about your level of interaction with patients? Do you have direct face to face contact or behind the scenes? In what ways do you support patients directly or behind the scenes? |
| 3. How often do you encounter patients who are in the protective custody of child welfare - or in 'foster care'? |
| 4. What would be your typical involvement for a patient in foster care? |
| 5. What kind of challenges do you face in your role when a patient is in foster care? |
| 6. Do you recall the information exchange platform IDENTITY? |
| 7. How did you learn about IDENTITY? |
| 8. It appears you have logged in at least once in the last 6 months, is that right? How often would you say you have used IDENTITY? |
| 9. Did you know that there are FYI In Protective Custody flags in Epic that will let you know when a child has a record in IDENTITY? Have you ever seen an FYI In Protective Custody flag in Epic in a patient's chart? If so, how did you use that information? Did that flag prompt you to access IDENTITY? Why or why not? |
| 10. How do you decide when to use IDENTITY? |
| 11. How do you typically use IDENTITY? i.e. look up one piece of information on one child? Look at all information on one? Look up multiple children at once? Another way? |
| 12. What information are you most interested in finding in IDENTITY? |
| 13. How does IDENTITY help you to do your job? |
| 14. Can you describe a time that information from IDENTITY changed your approach to a child? |
| 15. How might IDENTITY help improve patient outcomes? |
| 16. Can you describe a time that information from IDENTITY changed a child's outcome? |
| 17. Describe a time that you utilized IDENTITY (without any identifying information): |
| 18. What challenges do you face trying to access IDENTITY, if any? |
| 19. What would make IDENTITY more useful? |
| 20. Healthcare is provided by teams at [Institution name]. Right now, IDENTITY access is available for all team members. Which team members do you think should have access to IDENTITY? Should all team members be accessing IDENTITY during a patient encounter or only one/some? If one/some, how would you determine which ones? |
| 21. What privacy concerns do you think there are with the information contained in IDENTITY? Do you ever consider privacy when deciding whether to access IDENTITY? If so, how do you consider it? |
| 22. Any other feedback? |

| **Healthcare Staff Interview – IDENTITY Non-user** |
| --- |
| 1. Can you tell me a little about your current position/role at [Institution name]? What does a typical day look like for you? |
| 2. Can you tell me about your level of interaction with patients? Do you have direct face to face contact or behind the scenes? In what ways do you support patients directly or behind the scenes? |
| 3. How often do you encounter patients who are in the protective custody of child welfare - or in 'foster care'? |
| 4. What would be your typical involvement for a patient in foster care? |
| 5. What kind of challenges do you face in your role when a patient is in foster care? |
| 6. Have you ever heard of the IDENTITY data sharing platform? Our records indicate that you have not used IDENTITY in the past 6 months, is that right? Have you ever done the training for IDENTITY? |
| 7. Have you ever noticed a 'In Protective Custody FYI' flag in Epic? If so, have you ever read the reference to more information in IDENTITY? |
| 8. Were you ever curious to get more information in IDENTITY? Why or why not? |
| 9. Have you ever tried to access IDENTITY but had trouble? Please describe. |
| 10. What other strategies do you use to obtain information about patients in the child welfare system, such as caseworkers name or placement address? |
| ***Underwent brief IDENTITY Demonstration at this point*** |
| 11. Having now seen IDENTITY, do you think you might use it in the future? Why or why not? |
| 12. What information would you be most interested in finding in IDENTITY? |
| 13. How might IDENTITY help you to do your job? |
| 14. How might IDENTITY help improve patient outcomes? |
| 15. What challenges do you anticipate you may face trying to access IDENTITY, if any? |
| 16. After having seen IDENTITY, and thinking about how healthcare is provided by teams at [Institution name]. Which team members do you think should have access to IDENTITY? Should all team members be accessing IDENTITY during a patient encounter or only one/some? If one/some, how would you determine which ones? |
| 17. After having seen IDENTITY do you think there are any privacy concerns with the information contained in IDENTITY? If you do access IDENTITY in the future, would you consider privacy when deciding whether to access IDENTITY? If so, would you consider it? |
| 18. Any other comments? |

| **Caregiver Interview - Baseline** |
| --- |
| 1. How much health information do you currently have for the children in your home? |
| 2. Tell us about the relationship you have with the child |
| 3. At the time the child was placed with you, what did you know about their health? |
| a. Diagnosis information? |
| b. Allergies? |
| c. Medications? |
| d. Upcoming appointments? |
| e. Medical/Surgical history? |
| f. Other health information? |
| 4. Since the child was placed with you, what have you learned about their health? |
| a. How did you learn that information |
| b. When did you learn that information? |
| c. What was important that you learned? |
| d. What information was accurate? |
| e. What information was inaccurate? |
| f. What information was missing? |
| g. How did this impact care for the child? |
| h. How did this impact the medical care that you sought for the child? |
| ***Underwent brief IDENTITY Demonstration at this point*** |
| 5. How would having access to information earlier been beneficial to you in caring for the child? |
| 6. How does your access to information for the child compare to other children you currently or previously have cared for? |
| 7. How was the process of looking up the child in IDENTITY? |
| a. Was the information present? |
| b. How easy or hard was it to find? |
| 8. Now that you have reviewed the record for the child in IDENTITY, what information was new that you did not know about before today? |
| 9. What information included in the record for the child in IDENTITY would have been useful to you when the child was placed with you? |
| 10. How do you think you will use IDENTITY for the child now that you have access to it? |
| a. Call to schedule appointments? |
| b. Attend an already scheduled appointment? |
| c. Avoid an allergen? |
| d. Find out more about a medication? |
| e. Seek more information? |
| 11. What information are you still needing for the child, which may be missing from IDENTITY? |
| 12. Are there other features in IDENTITY that you would like to have? |
| 13. When do you think you will look up this child in IDENTITY again? What would prompt you to look at the child's record in IDENTITY again? |

| **Caregiver Interview – 30-day Follow-up** |
| --- |
| 1. Now that you have had access to the record for the child in IDENTITY for one month or longer, tell us about all the times (if any) when you used IDENTITY or when you could have used IDENTITY. |
| a. If you could have used IDENTITY but didn't, why not? |
| 2. What information included in the record for the child in IDENTITY was useful to you? |
| 3. What information are you still needing for the child, which may be missing from IDENTITY? |
| a. What information, if any, did you find from another source that was missing from IDENTITY? |
| 4. What descriptive information about the child's preferences or other information would you have wanted to see in IDENTITY, especially early in placement? |
| 5. What descriptive information about the child's preferences or other information would you be willing to enter into IDENTITY, especially in the weeks leading up to a placement transition, for example, reunification? |
| 6. Thanks so much for answering our questions about your experience using IDENTITY. Is there any other general feedback or information that you would like to share with us? |
